# Disagreements with implications: diverging discourses on the ethics of non-medical use of methylphenidate for performance enhancement

**DOI:** 10.1186/1472-6939-10-9

**Published:** 2009-07-06

**Authors:** Cynthia Forlini, Eric Racine

**Affiliations:** 1 Neuroethics Research Unit, Institut de recherches cliniques de Montréal, 110 avenue des Pins Ouest, Montréal, Québec, H2W 1R7, Canada; 2 Programmes de bioéthique, Université de Montréal, Montréal, Canada; 3 Department of Medicine and Department of Social and Preventive Medicine, Université de Montréal, Montréal, Canada; 4 Department of Neurology and Neurosurgery and Biomedical Ethics Unit, McGill University, Montréal, Canada

## Abstract

**Background:**

There is substantial evidence that methylphenidate (MPH; Ritalin), is being used by healthy university students for non-medical motives such as the improvement of concentration, alertness, and academic performance. The scope and potential consequences of the non-medical use of MPH upon healthcare and society bring about many points of view.

**Methods:**

To gain insight into key ethical and social issues on the non-medical use of MPH, we examined discourses in the print media, bioethics literature, and public health literature.

**Results:**

Our study identified three diverging paradigms with varying perspectives on the nature of performance enhancement. The beneficial effects of MPH on normal cognition were generally portrayed enthusiastically in the print media and bioethics discourses but supported by scant information on associated risks. Overall, we found a variety of perspectives regarding ethical, legal and social issues related to the non-medical use of MPH for performance enhancement and its impact upon social practices and institutions. The exception to this was public health discourse which took a strong stance against the non-medical use of MPH typically viewed as a form of prescription abuse or misuse. Wide-ranging recommendations for prevention of further non-medical use of MPH included legislation and increased public education.

**Conclusion:**

Some positive portrayals of the non-medical use of MPH for performance enhancement in the print media and bioethics discourses could entice further uses. Medicine and society need to prepare for more prevalent non-medical uses of neuropharmaceuticals by fostering better informed public debates.

## Background

The non-medical use of neuropharmaceuticals is generating substantial debates in medical and public health circles [1,2]. A key motive for this non-medical use of neuropharmaceuticals is the enhancement of cognitive function in healthy individuals beyond normal human capacity [3-5]. There is substantial evidence that methylphenidate (MPH; Ritalin), a drug prescribed to manage the symptoms of Attention Deficit/Hyperactivity Disorder (AD/HD) in children and adults, is being misused by healthy university students to improve concentration, alertness, and academic performance [6]. Much of this evidence has reported non-medical use of MPH in student populations but discussions of its ethical ramifications have also extended toward professional environments [7,8].

Methylphenidate is one of the most commonly used stimulants in the management of AD/HD symptoms [9,10]. This stimulant is associated with a high abuse potential [11-14] which has contributed to its classification as a scheduled substance in both the United States and Canada but continues to be used alongside newer slow release preparations. Academics and stakeholders have debated its prescription, and overprescription, making this stimulant a familiar name for healthcare providers and the public [15,16]. However, the debate on performance enhancement differs in that MPH is now being used for purposes unrelated to AD/HD. Furthermore, performance enhancement is distinct from "off-label" uses by physicians because performance enhancement uses are neither medically prescribed nor supervised. Recent studies have reported that this form of performance enhancement is a reality affecting North American university campuses but coverage in international print media suggests broader relevance [17-21]. Studies of prevalence rates show a range from 6.9% [22] to 35.3% [23] for prescription stimulant misuse in this student population. Closer examination of the motives behind the non-medical use of prescription stimulants yields rates from 3.2% up to 11% for the specific goals of improving concentration, alertness and academic performance [5]. Consequently, some scholarly ethical debates on the non-medical use of MPH have surfaced notably because: "In contrast to the other neurotechnologies [...] whose potential use for enhancement is still hypothetical, pharmacological enhancement has already begun" [3].

The scope and potential consequences of the non-medical use of MPH upon healthcare and society are wide-ranging and bring about many points of view and various discourses. In particular, media discourses can have important consequences on the practice of frontier health interventions and human welfare by shaping ethical debates and influencing public acceptance of neurotechnological innovation [24,25]. Accordingly, many fear that the public misunderstands the promises of neuroscience and their limitations [26,27] based on exaggerated or unbalanced media accounts. Consequently, it is important to examine the debate on pharmacological performance enhancement in the public sphere. This paper reports the results of a study which aimed to review and compare print media coverage with existing bioethics and public health discourses on the non-medical use of MPH for performance enhancement.

## Methods

This research aimed to analyze discourses on the non-medical use of MPH to enhance cognitive and academic performances. We examined discourses on the non-medical use of MPH in the print media (PM), bioethics literature (B), and public health literature (PH) based on previous work suggesting significant differences [5].

### Sampling

We generated the print media sample for this study using the *Factiva *and *LexisNexis Academic *databases consisting of full-text news, business, and law resources. We searched for English language newspaper articles published from 01/01/2000 to 11/14/2006 using guided news search options [28]. The start date of 2000 was chosen given the report of non-medical use of MPH in college students in the early 2000s [29]. Multiple keyword searches were used to identify articles discussing the non-medical use of MPH (Table 1). Keywords were searched in headline, lead paragraph(s) and general news (major papers) in *Factiva *and *LexisNexis Academic *databases. Bioethics and public health publications were sought using standard databases. All articles yielded by the searches were carefully examined for relevance, the key criteria being the discussion of the non-medical use of MPH. Individual articles were the sampling units.

**Table 1:** Generation of sample for analysis of discourses on the non-medical use of methylphenidate.

Discourse	Databases	Keywords	Articles (n)
Print media	Lexis-Nexis Academic Factiva	"Ritalin" "methylphenidate" "smart drugs" "Ritalin & study aid" "cognitive enhancement" "neuroethics" "stimulant abuse" "non-medical use" "illicit use"	20*^†^
		
Bioethics literature	PubMed (bioethics limit) Expanded Academic		14
		
Public health literature	PubMed Expanded Academic		7

* We found a single article repeated four times (N = 23 articles) but kept the twenty distinct articles for analysis (except for the headline analysis since all 23 headlines were distinct).

^† ^Articles originated from the USA (n = 11), UK (n = 6), Australia (n = 2) and Canada (n = 1).

### Coding

The content of all articles was coded systematically using the QSR NVivo 7 software (Doncaster, Australia). The inductively-generated coding guide and grid were inspired by previous content analyses of print media [28,30,31] but adapted to our object of research. Adaptation of the coding guide was pursued through multiple rounds of piloting and test coding on a sub-sample of print media articles to ensure validity and robustness [32]. Key codes were derived through an inductive process in which previously used coding categories for content analysis [31] were refined and adjusted to the context of non-medical use of MPH. This coding guide defined each category and provided both an explicit (upper limit) and implicit (lower limit) example of what each code could be applied to. After the initial coding of the whole sample by one member of the research team, two other members of the research team reviewed the content of each category to ensure reliability of coding by consensus and ensure that each code was within the limits established by the coding guide. The final coding structure included four major areas (Additional file 1): (1) description of the non-medical use of MPH; (2) workings and effects of MPH, including positive and negative effects associated with non-medical use of MPH; (3) description of ethical, social, and legal issues associated with non-medical use of MPH and (4) recommendations for the prevention of the non-medical use of MPH. Coding of the ethical, legal and social content was furthered by determining if the coded statements affirmed, negated, or remained neutral or ambivalent regarding the issue at stake.

Given our goal of also examining different ethical, social and legal issues surrounding the non-medical use of MPH, we report the frequency and distribution of various codes. Data is reported to illustrate the nature of statements found within discourses and contrast discourses on the non-medical use of MPH.

## Results

### Portrayal of non-medical uses of MPH in different discourses

We first examined how the non-medical use of MPH for performance enhancement was portrayed in the media in comparison to scholarly bioethics and public health discourses. We found that a wide array of terms was used in the print media [18-21,33-38], many of which conferred a sense of familiarity and efficacy (e.g., "study aid", "study tool") regarding this form of MPH use (Table 2).

**Table 2:** Occurrences of lay designations of methylphenidate used non-medically for performance enhancement in the print media

Lay designation	Occurrences
"study aid" [37]	9

"brain steroid" [18]	4

"smart drug(s)" [21]	4

"vitamin R" [37]	4

"poor man's cocaine" [33]	3

"study tool(s)" [18]	2

"wonder drug" [38]	2

"new chemical aid" [18]	1

"smart pill(s)" [35]	1

"cramming drug" [34]	1

"academic steroids" [37]	1

"steroids of academia" [20]	1

"legal speed" [36]	1

"kiddie speed" [19]	1

In the media, typical statements described the non-medical use of MPH for performance enhancement as a lifestyle choice or a form of illicit street drug (e.g., "better living through chemistry") [18,39] while the formal term of "cognitive enhancement" was found almost exclusively in bioethics discourse [40]. Public health discourse described this practice negatively as a form of illicit prescription drug misuse or abuse [41] (Table 3). Headlines used in different discourses reflected these divergent frameworks (Table 3). In addition to the reported non-medical uses for performance enhancement (20 PM; 14 B; 7 PH), which was a selection criteria, other uses of MPH were generally discussed including medical uses to treat AD/HD (16 PM; 5 B; 5 PH) and recreational uses (6 PM; 1 B; 7 PH).

**Table 3:** Portrayal of the non-medical use of methylphenidate in print media, bioethics and public health supported by examples of article headlines as well as the occurrence of reported risks and benefits indicated in parentheses (n).

*Print media*	
*Portrayal*	“Lifestyle choice”: “better living through chemistry” [39]; “short cut in learning”; “new kind of drug abuse” [18].

*Examples of headlines*	"Students taking danger drug to help with exams"; " 'Smart pills' are on the rise. But is taking them wise?"; "New campus high: Illicit prescription drugs".

Reported risks*	Physiological addiction (8); palpitations (7); psychological addiction (6); heart attack (5); unspecified cardiac risks (4); loss of appetite (4); hallucinations (4); stroke (2); tremors (2); increase in blood pressure (2); weight loss (2); vomiting (2); dizziness (2); seizures (2); withdrawal symptoms (2); require increasing amounts of drug (1); cardiac arrhythmia (1); overdose (1); changes in brain cell chemistry (1); fatigue (1); death (1); dry mouth (1).

*Reported benefits*^†^	Boost concentration (8); increase focus (7); increase energy (3); increase alertness (1); reduce appetite (1); eliminate jitters (1); filter out distractions (1); increase motivation (1); accumulate more information in less time (1); increase confidence (1); increase organization (1); increase retention of information (1); think more rationally (1); general feeling of well-being (1); make you feel smarter (1); make mundane tasks seem fun (1); enhance studying (1); do work faster (1); maintain high performance level (1); boost brain activity (1).

*Bioethics*	

*Portrayal*	*"Cognitive enhancement"*: " 'neuroenhancement' (...) This term includes the use of drugs and other interventions to modify brain processes with the aim of enhancing memory, mood and attention in people who are not impaired by illness or disorder"[40].

*Examples of headlines*	"Neurocognitive enhancement: what can we do and what should we do?" and "Cosmetic neurology: The controversy over enhancing movement, mentation, and mood".

Reported risks*	Addiction (3); toxicity (1).

*Reported benefits*^†^	Improve attention (4); improve memory (4); improve performance (2); increase focus (1); improve concentration (1); improve planning (1); think faster (1); stabilize mood (1); promote creativity (1).

*Public health*	

*Portrayal*	*"Abuse", "misuse", "illicit drug use"*: "Ritalin (Novartis Pharmaceuticals Corp., East Hanover, NJ) has received the most attention in medical literature, little information is available regarding which specific stimulants are used illicitly by college students" [41].

*Examples of headlines*	"Student perceptions of methylphenidate abuse at a public liberal arts college" and "Stimulant medication use, misuse, and abuse in an undergraduate and graduate student sample".

Reported risks*	Addiction (2); cardiovascular implications (1); withdrawal symptoms (1); increase in blood pressure (1); headache (1); overdose (1); blocking veins if injected/snorted (1); panic episodes (1); aggressive behavior (1); suicidal or homicidal tendencies (1).

*Reported benefits*^†^	Decreases fatigue (2); increases energy (1); increases dopaminergic activity (1); maintain high performance level (1); increase alertness (1).

*Coded as physiological/psychological negative effects

^†^Coded as physiological/psychological positive effects

In terms of risk and benefit statements, the risk of addiction was present in all discourses while the print media presented a wider variety of risks. However, in comparison to the prescribing information provided by Novartis for MPH in the US, Canada, UK, and Australia [42-45] (where the articles originated), most of these risks were common risks and few of the uncommon and rare side effects were featured.

Analyzed articles typically described: (1) who is using MPH non-medically (e.g., college students; 18 PM; 8 B; 6 PH); (2) when MPH is used non-medically (e.g., during final exams; 13 PM; 0 B; 3 PH); (3) where MPH is used non-medically (e.g., college campuses and high schools; 11 PM; 5 B; 4 PH). Details were also reported, notably in print media, on how students where securing MPH for non-medical uses, i.e., by buying pills from other students (14 PM; 1 B; 3 PH); by feigning symptoms of ADHD (5 PM; 0 B; 2 PH); through black markets (5 PM; 0 B; 1 PH); through Internet pharmacies (4 PM; 0 B; 0 PH) and by stealing pills (3 PM; 0 B; 1 PH).

The extent and social acceptance of non-medical uses of MPH was described in divergent ways particularly in the print media. We found contrasting statements that this practice was: (1) "accepted" (6 PM; 0 B; 1 PH); (2) "frequent" and "widespread" (16 PM; 8 B; 5 PH); (3) the subject of ambivalent opinions (6 PM; 0 B; 0 PH); (4) "debatable" and "concerning" (10 PM; 1 B; 3 PH); and (5) rare and anecdotal (6 PM; 0 B; 0 PH). Bioethics discourse alluded to the treatment-enhancement dichotomy, often judged to be blurry or misleading (2 PM; 9 B; 0 PH) to understand the implications of non-medical use of MPH.

### Ethical, legal and social issues of non-medical use of MPH

There were generally wide-ranging views on the ethical, legal and social issues related to the non-medical use of MPH. Table 4 provides an overview of the frequency and distribution of ethical, legal and social issues within the discourses examined. The issues most discussed across discourses were social integration and acceptability (e.g., "Ritalin makes repetitive, boring tasks like cleaning your room seem fun," (...) "I equate it in my mind with a really strong cup of coffee" [46]), social meaning (e.g., "Ritalin acts as a quick fix for problems that are the product of the rapid-fire culture and the hurried society in which we live" [17]), and safety (e.g., "Heiligenstein says that part of the problem is a perception that prescription drugs, as opposed to "street" drugs, are safe because they have been officially approved" [37]).

**Table 4:** Frequency and distribution of ethical, social and legal issues associated with the non-medical use of methylphenidate (MPH) for performance enhancement in print media (PM), bioethics (B) and public health (PH) discourses.

*Ethical, legal and social issues*	*Affirmation*				*Negation*				*Total Frequency*
		
	*PM*	*B*	*PH*	*Total*	*PM*	*B*	*PH*	*Total*	
*Social integration and acceptability*	11	13	-	24	5	-	-	5	29

*Social meaning*	11	9	5	25	1	2	-	3	28

*Unsafe*	2	11	2	15	3	2	2	7	22

*Abuse*	11	1	5	17	1	1	1	3	20

*Cheating*	5	8	-	13	3	3	-	6	19

*Inauthenticity, identity and personhood*	1	12	-	13	-	5	-	5	18

*Injustice and inequalities*	-	10	-	10	-	6	-	6	16

*Overprescription*	9	2	4	15	1	-	-	1	16

*Lack of autonomy, individual choice and informed consent*	-	10	-	10	-	5	-	5	15

*Illegality*	8	2	-	10	3	1	-	4	14

*Commercialization*	1	7	-	8	-	1	-	1	9

*Inefficacy*	2	4	1	7	-	-	-	-	7

Bioethics discourse was comprehensive in its coverage of ethical and social issues generally representing both affirmations and negations, i.e., statements that an issue was important and rebuttal statements of it. Some issues were absent from print media and public health discourses: inauthenticity, identity and personhood; lack of autonomy, individual choice and informed consent; injustice and inequalities. Despite many negations that MPH is unsafe, none of the discourses expressed the belief that we had reliable evidence of efficacy to support such statements. In fact, we only found seven negations i.e., there is no reliable data on the cognitive enhancement effects of MPH. All three discourses also seemed to converge, save for one negation in the print media, on the topic that MPH was overprescribed.

Generally, the ethical, legal and social issues related to the non-medical use of MPH typically cautioned against more widespread use. For example, issues like abuse; commercialization; illegality; inauthenticity, identity and personhood; lack of autonomy, individual choice, and informed consent as well as concerns regarding safety were more often affirmed than negated. However, we observed marked ambivalence with regard to other issues. For example, on the topic of enhancement and cheating some sources stated that the non-medical use of MPH creates an unfair playing field (e.g., "Some students who don't use the drug say their pill-popping classmates have an unfair edge and consider use of the pills a form of cheating" [47]) while others consider it as fair as private tutors (e.g., "You deserve to win a Nobel Prize if you discover the cure for cancer, whether or not you do so with the aid of cognitive enhancement drugs" [48]). We found a similar situation with respect to the issue of injustice and inequalities. Some sources maintained that cognitive enhancement cannot erase the "disparities created by natural talent and luck" [48] but others argued that "Unequal access is generally not grounds for prohibiting neurocognitive enhancement, any more than it is grounds for prohibiting other types of enhancement, such as private tutoring or cosmetic surgery that are enjoyed mainly by the wealthy" [3].

### Recommendations for the non-medical use of MPH

Many types of solutions were proposed to deal with non-medical use of MPH [3,17,18,33,34,37,38,40,41,47-53] (Table 5). Bioethics discourse called for a range of measures. These suggestions included restrictive legislation on MPH and other potential performance enhancing drugs in healthy people by criminalizing non-approved uses. Contrasting recommendations to facilitate access to these drugs (e.g., government subsidies for those who cannot afford the drugs) were also presented. The print media and public health discourses promoted changing the habits of healthcare professionals in diagnosis and prescription and also informing students and university staff about the misuse of prescription drugs and its risks.

**Table 5:** Proposed recommendations to prevent non-medical use of methylphenidate (MPH) and challenges associated with prevention.

*A. Proposed recommendations to prevent non-medical use of MPH*		
Print media	Bioethics	Public health

Diagnosing ADHD more carefully [37,38,50] Supervising of students with stimulant prescriptions [33,47] Teaching students effective study skills and stress management [17,37] Informing students and staff of the dangers of abusing prescription drugs [18,33,34,37]	Criminalizing non-approved uses of medications [3,48] Prohibiting prescription of drugs for lifestyle purposes by doctors [48] Obliging manufacturers to declare safety data for unapproved uses to the FDA [48] Subsidizing cognition enhancing drugs to allow equal access [40,48] Establishing a "ceiling" as the maximum cognitive enhancement permissible [48,53]	Ensuring prescription compliance and responsible prescription practices [49,52] Prescribing preparations that are less easily abused [41,51] Identifying persons who are liable to abuse medication [49] Educating healthcare providers dealing with university populations as to the abuse potential of stimulants [51,52]

*B. Identified challenges in the prevention of non-medical use of MPH*		

Print media	Bioethics	Public health

Logistical problems of enforcing a ban [37,38,54] Perceived safety of MPH makes convincing students about its dangers more difficult [35,37] Abundance of MPH in healthcare system [50] Misuse of MPH bypasses traditional sources of information on indications and risks when taking a prescription medication [37]	Difficult to propose a ban on cognitive enhancers because of their routine use in treatment [40,48,53] Ban is liable to encourage a black market and be just as coercive as social pressure [40,48,53] FDA has little experience in assessing social cost/benefit of a drug and thus is unfit to take charge of such regulation [53]	None identified

Several challenges were highlighted regarding the prevention of non-medical use of MPH [35,37,38,40,48,50,53,54] (Table 5). These included the logistical complexity and legitimacy of enforcing a ban and the detrimental impact of a ban on patients who need a drug to function. The most emphasized challenge was the sense of security that individuals have with regard to prescription drugs, because they are approved by governmental health agencies.

## Discussion

This study examined discourses on the non-medical use of MPH by college students in the print media, bioethics literature, and public health literature. We found that there were three distinct paradigms used to describe the non-medical use of MPH. The "lifestyle choice" framework expressed in the print media generally reflected the opinion that the uses of neuropharmaceuticals for self-improvement is a laudable goal and a personal choice. The "prescription drug abuse" framework found in the public health discourse views the non-medical use of neuropharmaceuticals as illicit and a public health problem. The "cognitive enhancement" framework in bioethics discourse focuses on the ethical issues arising from presumed benefits of non-medical use of neuropharmaceuticals by healthy individuals. These paradigms were reflected notably in the headline content across discourses and the statements used to describe the non-medicinal use of MPH itself (Table 3). The lifestyle paradigm is also well illustrated in the print media's use of lay designations (Table 2) and the enthusiastic terms used to describe its potential beneficial enhancement effects of non-medical use of MPH (Table 3). We observed diverging claims about the frequency and acceptability of such MPH use. The print media provided overall detailed descriptions of whom, where, and when MPH was used non-medically and also how students were procuring it. The ethical discussion surrounding the non-medical use of MPH was without surprise more comprehensive in the bioethics literature. Overall, discussion of ethics was wide-ranging and only in the public health literature was there a clear stand against non-medical uses of MPH. Issues most frequently discussed concerned social aspects as well as safety considerations of the non-medical use of MPH for performance enhancement. Major areas of debate included whether performance enhancement with MPH was considered cheating as well as whether the phenomenon created injustices and inequalities. Discourses converged on the topics of overprescription of MPH and lack of reliable scientific data for the enhancement effects of MPH. Recommendations ranging from calls for legislation to increased public education were identified in all three sources of discourse but challenges to these recommendations were only identified and discussed in the print media and in the bioethics literature (Table 5).

### Limitations

As with most qualitative research and discourse analyses, some aspects of our study limit the generalization of the results. First, the small sample size and limited sample composition, in spite of broad searches and the use of multiple databases, are not exhaustive of all discourses on non-medical use of MPH. The results of this small study should accordingly be viewed as a preliminary step to fulfill this larger goal. Second, the scope of the study was limited to a few countries, mostly because of the available sources of the literature. Third, the specific case of the non-medical use of MPH was examined even though there are other drugs that are used in similar ways. However, this choice is supported by the draw of MPH for performance enhancement as reported in a recent survey published in Nature [48] and its well established use by university students [6]. Fourth, the reported statements in the print media articles are an amalgamation of opinions from people interviewed by journalists and do not necessarily reflect the opinions of journalists. Accordingly, the print media content should be viewed as what was available to the public through this channel rather than the voice of journalists per se.

### Disagreements between paradigms could have important healthcare, ethics, and social implications and consequences

The dissonance we observed between paradigms used to describe and evaluate the non-medical use of MPH for performance enhancement could have profound healthcare, ethics and social implications and consequences. Each paradigm carries forward a distinct view of the acceptability of MPH non-medical use. Speaking of a "lifestyle choice", a "cognitive enhancement", or a "prescription misuse" matters for scholarly biomedical ethics, public debate, healthcare and public policy. A major source of disagreement and concern is the unbalanced presentation of the potential positive and negative effects of MPH across discourses. In the print media especially, a fair number of potential adverse effects (Table 3) are mentioned but most often without qualification or quantification. In contrast to the risks, the positive effects are discussed using sensational terms like "wonder drug" or "smart drug" (Table 2). As suggested by Rajczi [55], such enthusiastic discourses about new technologies can arise from society's assumption that they are intrinsically valuable but can be independent of the scientific evidence. Furthermore, Lanni *et al*. argue that: "From a pharmacology point of view the fact that a drug is clinically used to treat an attention disorder or a cognitive problem does not necessarily mean that a high level of the relevant molecule would produce a high performance in a normal individual" [56]. To date, the benefits of cognitive enhancement for healthy individuals appear to be based on media reports and a few scientific studies. However, the alleged efficacy of cognitive enhancers is an important area of disagreement and diverging perspectives.

Before rallying behind cognitive enhancement and most definitely before any kind of regulation or approval is put forward, current scientific data must be assessed and interpreted carefully beyond general assumptions inherent in terms like "cognitive enhancers" and "smart drugs". Contrary to some implicit assumptions found in bioethics and media discourses, there are actually only a few studies on the enhancement effects of cognitive enhancers on healthy individuals. The findings of these studies may in fact be limited in their potential to be generalized and support favorable opinions toward the cognitive enhancement of healthy individuals. For example, Elliott *et al*.'s often-cited, double-blind, placebo-controlled study showed that methylphenidate positively impacted performance on spatial working memory and planning but not on attention and fluency tasks. However, the results also showed that methylphenidate did not enhance performance tasks that were already learned [57]. The study conducted by Mehta *et al*. investigated changes in regional cerebral blood flow as an indication that methylphenidate enhances spatial working memory [58]. Barch *et al*. obtained results similar to the findings of Elliott *et al*. and Mehta *et al*. that amphetamine enhanced spatial working memory [59]. In contrast to the three previous studies, Bray *et al*. reported that methylphenidate does not enhance the cognition of sleep-deprived individuals [60]. Farah *et al*. recently examined the effect of Adderall upon creativity, a component of cognition stimulants are suspected of stifling, in a double-blind, placebo-controlled trial [61]. They found that the drug indeed enhanced creativity on specific tasks but the amount of enhancement depended upon the baseline performance of individuals: lower-performing individuals were more enhanced than high-performers. The conflicting and fragmented results of these few studies currently provide limited support for the enthusiastic portrayals of cognitive enhancement.

To date, the studies supporting enthusiastic media and bioethics discourses do not reflect the reality of research on MPH-based enhancement. Several aspects of early research need to be further examined and reproduced. First, generalizing results at this point is imprudent given the small and homogeneous cohorts involved. Samples in these studies range from ten to twenty-eight participants and most of them are young healthy males. Current parameters cannot account for variable efficacy in individuals which would result in certain types of individuals being unable to enhance themselves thus perpetuating the debates on justice because of unequal effects. Second, the specific experimental nature of the tasks used for research does not necessarily reflect real-world performance in complex contexts. Further, Farah *et al*.'s results show that there may be an "enhancement ceiling" for certain types of individuals or tasks. Third, there is limited understanding of the long-term effects of MPH and other drugs used for enhancement. Presently, for treatment with MPH lasting more than four weeks, it is strongly recommended that the treating physician regularly reevaluate the necessity for the prescription for MPH [62]. The survey on cognitive enhancement conducted by *Nature *revealed that respondents used cognitive enhancers on daily, weekly and monthly bases in almost even proportions [63] which indicated that enhancement may become a habit. In the laboratory setting, MPH has been shown to have an abuse potential under certain conditions [11-14] that may result in addiction with regular use of cognitive enhancers. In contrast to enthusiastic media and bioethics discourses, these observations have the potential to show that non-medical use of MPH may be less valuable than some expect because of the scientific and medical limitations of the drug effects.

The reported positive effects in the print media are largely based on anecdotes and are typically not adequately contrasted with scientific data about the effects of MPH on the healthy brain. Given these features, some enthusiastic interpretations found in print media as well as bioethics discourse could contribute to the unintended dissemination of a poorly understood non-medical use of MPH for performance enhancement. However, if public health discourses prematurely condemn this practice as a form of drug abuse, future public health strategies risk being ill-equipped to tackle the enthusiasm and interest for cognitive enhancers found in other discourses and perhaps in the public.

### Bioethics and the print media need to sustain public information and socially-informed public debate

Bioethics discourse and, to some extent, the print media, contained extensive discussions on the ethics of the non-medical use of MPH. Nonetheless, our results suggest that the coverage of the phenomenon in these discourses brings about sources of confusion. For instance, there is a wide range of uncertain claims about the prevalence and risks of the practice. The print media, in particular, conveys in many respects more sociological details and context (e.g., who, how, when and where) regarding the non-medical use of MPH for performance enhancement. However, this may have unexpected consequences such as increasing the prevalence of the practice. In fact, the combination of consumption details and student testimonials with positive portrayals of the performance enhancement potential of MPH in the print media may incite individuals to engage in the practice.

Our results also suggest that using MPH for enhancement is already considered an integral part of social practice in some discourses. They show that some media reports consider MPH comparable to "traditional" methods of enhancement that are acceptable such as consuming caffeine. Combined with praise of MPH as a "miracle drug", this perceived social integration can build additional fervor for cognitive enhancement. This perception may be a reflection of the social context in which cognitive enhancement is emerging and would need to be captured in the bioethics discussion. Recent work on the medicalization of sleep and non-medical uses of modafinil has linked the acceptability of pharmacological enhancements to the context of use [64]. In his book *Listening to Prozac*, Kramer postulates that: "The operational definition of wellness must be in relation to the demands and goals of society, here and now" [65]. Not only does social context first modulate the definition of health on the treatment-enhancement spectrum but it also affects other issues. For example, coercion of individuals to use cognitive enhancers is often cited as an issue in the literature [3,40,48]. It was also identified as an important issue in our present discourse analysis. However, the specific nature of coercion remains vague without an indication of what causes this coercion and what stakeholders stand to gain. Both of these aspects are partly shaped by social context and are important to consider in ethical discussion and policy. However, critical outlooks on social context could pose a real challenge given the favorable opinions of influential bioethicists who emphasize the role of personal choice and individual rights in the choice to enhance cognition [66]. Even though we found some aspects of media and bioethics discourses to be favorable and enthusiastic, they can also serve as important venues to voice attitudes and informing the public of the risks associated with neuropharmaceuticals.

The nature of information disseminated to the public is also of the utmost importance. There are potentially some valuable lessons about reporting on non-medical uses of MPH and other pharmaceuticals that can be gleaned from the guidelines put forth by the Australian Press Council. The Council's guidelines relate to journalistic reporting on illicit drug use (drug addiction). They recommend avoidance of reporting "stories that might excite the interest of young people in drug experimentation, including the naming of dangerous drugs". The recommendations for reporting on addictive drugs also state that "the harmful effects of any particular drug should not be exaggerated or minimized"; and that we should "avoid detailed accounts of consumption methods, even though many young people are generally familiar with them." The recommendations also "guard against any reporting which might encourage readers' experimentation with a drug, for example highlighting the 'glamour' of the dangers involved" [67]. Though using neuropharmaceuticals to enhance cognitive performance happens in a different context than illicit drug abuse they do share some similarities. The thinking behind these guidelines could translate into avoidance of narratives and salient practices related to non-medical MPH by students as well as other forms of non-medical use of prescription drugs. This represents a strong stance that could appear paternalistic and an interference with good reporting practices but the onus of responsible reporting does not lie exclusively upon journalists. There are various stakeholders that could positively contribute to the ethical deliberation on cognitive enhancement and several reasons why their input would be beneficial. For instance, healthcare providers being interviewed on this topic may want to be vigilant about the opinions they express to journalists about non-medical practices especially regarding risks and benefits. For example, we found a clinical psychiatrist that was quoted as saying "Caffeine is fine. This is better (...) Students are able to accumulate more information in a shorter time frame. These drugs keep you awake longer. They minimize fatigue and help maintain a high performance level" [47]. Perhaps healthcare professionals should be careful with such public comments on non-medical uses of pharmaceuticals. Public health agencies must also be aware of enthusiastic media reports if they want to counterbalance unwarranted messages in the media and better inform the public and stakeholders. These are some initial venues to explore to improve the commitment to public information and informed debate on non-medical uses of prescription drugs for enhancement purposes.

Beyond the improvement of journalistic practices and the responsible involvement of healthcare providers in stories about the non-medical use of MPH, broader public engagement needs further consideration given the stakes. Though the decision to use a neuropharmaceutical for cognitive enhancement may be up to the individual, the effects of the enhancement loom much larger. The choices of individuals may impact collective behaviors which clearly makes the subject of cognitive enhancement a public matter. However, public engagement is more than just informing the public. It is also listening to public voices. Racine *et al*. have proposed a model where inquiry and debate on a given scientific development is at the center of multi-dimensional communication between the scientific community, humanities and social science, the media as well as the public and stakeholders [30]. With this model, knowledge about advances in neuroscience (or other branches of science) does not end when it reaches the public. Instead, public perceptions and opinion are fed back up the chain of knowledge to instruct the scientific community about what the public has understood about its work. Possibly the most interesting aspect of this model is that there is also dialogue between other stakeholders. The multi-directional aspect of models like these pave the way to engaging stakeholders in ethical discussion of cognitive enhancement to provide a richer and broader spectrum of perspectives.

### Medicine, healthcare, and society need to prepare for broader and more prevalent non medical uses of pharmaceuticals

In our study, public health discourses on enhancement raised many concerns about the non-medical use of MPH for performance improvement because of its potential health consequences. The prevalence of this practice with MPH, which ranges from 3.2% to 11%, [51,52] is worrisome from a healthcare perspective since it involves the use of a controlled substance by individuals outside of the intended clinical context. This trend has the potential to pave the way for the general acceptability of non-medical uses of other pharmaceuticals. Accordingly, societies could be faced with serious public health challenges before the ethics of this practice is properly discerned and publicly debated. The 2007 report on enhancement from the British Medical Association discussed whether a role for public health was timely but did not conclude on the subject [1]. We did note that wide ranging solutions were suggested to prevent the expansion of the non-medical use of MPH for enhancement purposes (Table 5). Development of legislation on non-medical uses and distribution of prescription medications as well as the education of healthcare professionals and the public about the dangers of misusing prescriptions were common suggestions. It should be noted that possession of a prescription drug without permission and trafficking can have criminal implications but this has not discouraged non-medical uses for performance enhancement. Furthermore, students who feign symptoms of AD/HD can acquire legitimate prescriptions for non-medical motives.

This situation mirrors to some extent the widespread illegal provision of human growth hormone (hGh) in the US [68]. The recent recommendations in Olshanky and Pers' paper regarding this practice focus mostly on the illegal distribution of hGh by manufacturers but also highlighted the ethical responsibilities of healthcare professionals. Though the stakeholders in cognitive enhancement with neuropharmaceuticals are different than in hGh, there appear to be similar problems with fraudulent sales online and trafficking of MPH among students [37,63]. Prevention of these types of distribution, stricter prescription practices, better patient prescription compliance and effective, balanced information to the public could help decrease prevalence and social integration of the practice in the absence of medical, social, and ethical consensus about its acceptability. Regulatory bodies and policy makers could begin examining the hGh recommendations as well as their associated challenges to model potential action with regard to the emerging practice of the non-medical use of MPH. However, before any new policies are made there clearly needs to be a broader debate on the non-medical uses of neuropharmaceuticals in order to sort through ethical and social issues.

At this time many consider that the non-medical use of MPH for cognitive enhancement happens outside the confines of medicine. Physicians and allied healthcare professionals could be eased out of their role as "gatekeepers" to these types of drugs as suggested by Chatterjee [69]. Unfortunately, it is not well-known if all healthcare providers are aware of the prevalence of the non-medical use of methylphenidate and other neuropharmaceuticals for enhancement or if they would feel concerned at all. Data from the US National Institutes of Health shows that over 40% of healthcare providers have difficulty addressing the subject of prescription abuse with their patients. For physicians, the subject of prescription abuse appears to be even more difficult to tackle than stigmatized conditions like depression and alcoholism [70]. A survey of general practitioners on the subject of enhancement with pharmaceuticals in the *Scandinavian Journal of Public Health *indicated that general practitioners were not open to the use of prescriptions for enhancement purposes [71]. These results suggest that healthcare providers are not fully aware of the prevalence of the non-medical use of pharmaceuticals for enhancement and that they potentially perceive enhancement to be outside the boundaries of medicine and perhaps out of their professional role. Even though some areas of medicine may consider cognitive enhancement to be outside of the realm of healthcare, some aspects of the phenomenon may call for public health interventions. This creates a vexing situation where healthcare providers' view of cognitive enhancement as a non-medical practice could curtail consideration of its public health implications. However, even when the pills are obtained on the black market they still, most likely, were paid for by some patient's health insurance. Consequently, the use of medical personnel and financial resources for cognitive enhancement of healthy individuals could be putting a strain on healthcare systems. Viewed in this light, users of cognitive enhancers may actually be inviting public health action and policy by consuming medical resources.

Currently, public health action for the prevention of the non-medical use of pharmaceuticals for cognitive enhancement faces some important challenges. First, it is important that healthcare professionals become more aware of the non-medical use of pharmaceuticals for cognitive enhancement. This would likely make them more comfortable discussing the topic. Second, the burden of responsible management of prescriptions may well fall on healthcare professionals and patients alike but the reality is that healthcare providers have little or no control over what is done with prescriptions when patients leave their offices. Public health information campaigns trying to prevent prescription misuse could perhaps more directly target enhancement uses of prescriptions. Lastly, in raising awareness among the public with regard to cognitive enhancement public health faces a possible conflict of values. On one hand, public health action aims to prevent practices that are potentially harmful to the public's health like taking a pharmaceutical without a prescription. On the other hand, raising awareness may inadvertently promote forms of cognitive enhancement of healthy individuals. Public health interventions will need to carefully consider how to play a role in cognitive enhancement.

## Conclusion

This study examined diverging discourses on the non-medical use of methylphenidate for performance enhancement. Our research yielded thought provoking results on the respective portrayals of such use of MPH in print media, bioethics and public health discourses. We have highlighted some of the potential implications of current discourses, notably the enthusiastic portrayals of the non-medical use of MPH in the print media and bioethics discourses despite scarce scientific data supporting its enhancement effects. In light of the diverging views on ethics and enhancement but the increasing salience of debates on "cognitive enhancers", we believe that public action and more accurate representation of current scientific evidence are needed. Engagement of stakeholders and the general public could enrich the discussion and provide broader perspectives on the non-medical use of MPH.

## Competing interests

The authors declare that they have no competing interests.

## Authors' contributions

CF contributed to the design of the study, generated the samples, performed data analysis and data interpretation, drafted and revised the manuscript. ER designed the study, participated in the data analysis and data interpretation, helped draft and revise the manuscript. Both authors read and approved the final manuscript.

## Pre-publication history

The pre-publication history for this paper can be accessed here:



## Supplementary Material

Additional file 1 **Coding structure used to analyze media, bioethics, and public health discourses on the non-medical use of methylphenidate (MPH).** Click here for file

## References

[B1] British Medical Association (2007). Boosting your brainpower: ethical aspects of cognitive enhancement.

[B2] Canadian Centre on Substance Abuse (2007). Prescription drug abuse FAQs.

[B3] Farah MJ, Illes J, Cook-Deegan R, Gardner H, Kandel E, King P, Parens E, Sahakian B, Wolpe PR (2004). Neurocognitive enhancement: what can we do and what should we do?. Nature Reviews Neuroscience.

[B4] Flower R (2004). Lifestyle drugs: pharmacology and the social agenda. Trends in Pharmacological Science.

[B5] Racine E, Forlini C (2008). Cognitive enhancement, lifestyle choice or misuse of prescription drugs? Ethical blindspots in current debates. Neuroethics.

[B6] Wilens TE, Adler LA, Adams J, Sgambati S, Rotrosen J, Sawtelle R, Utzinger L, Fusillo S (2008). Misuse and diversion of stimulants prescribed for ADHD: a systematic review of the literature. Journal of the American Academy of Child and Adolescent Psychiatry.

[B7] Appel JM (2008). When the boss turns pusher: a proposal for employee protections in the age of cosmetic neurology. Journal of Medical Ethics.

[B8] Warren OJ, Leff DR, Athanasiou T, Kennard C, Darzi A (2008). The Neurocognitive Enhancement of Surgeons: an ethical perspective. J Surg Res.

[B9] Wolraich ML, Wibbelsman CJ, Brown TE, Evans SW, Gotlieb EM, Knight JR, Ross EC, Shubiner HH, Wender EH, Wilens T (2005). Attention-deficit/hyperactivity disorder among adolescents: a review of the diagnosis, treatment, and clinical implications. Pediatrics.

[B10] Butcher J (2003). Cognitive enhancement raises ethical concerns. Academics urge pre-emptive debate on neurotechnologies. Lancet.

[B11] Compton WM, Volkow ND (2006). Abuse of prescription drugs and the risk of addiction. Drug and Alcohol Dependence.

[B12] Kollins SH, MacDonald EK, Rush CR (2001). Assessing the abuse potential of methylphenidate in nonhuman and human subjects: a review. Pharmacology, Biochemistry and Behavior.

[B13] Volkow ND, Ding YS, Fowler JS, Wang GJ, Logan J, Gatley JS, Dewey S, Ashby C, Liebermann J, Hitzemann R, Wolf AP (1995). Is methylphenidate like cocaine? Studies on their pharmacokinetics and distribution in the human brain. Archives of General Psychiatry.

[B14] Volkow ND, Swanson JM (2003). Variables that affect the clinical use and abuse of methylphenidate in the treatment of ADHD. American Journal of Psychiatry.

[B15] Diller LH (1996). The run on Ritalin. Attention deficit disorder and stimulant treatment in the 1990s. Hastings Centre Report.

[B16] Singh I (2008). Beyond polemics: science and ethics of ADHD. Nature Reviews Neuroscience.

[B17] Bayer K (2004). Students taking danger drug to help with exams.

[B18] Laurance J (2003). Abuse hits students looking for an exam kick.

[B19] Lawrence J (2004). 'Kiddie speed' for exam boost.

[B20] O'Regan N (2005). Want to be clever? Don't take 'smart drugs'.

[B21] Ross S (2006). Students turn to smart drugs for exam help.

[B22] McCabe SE, Knight JR, Teter CJ, Wechsler H (2005). Non-medical use of prescription stimulants among US college students: prevalence and correlates from a national survey. Addiction.

[B23] Graff Low K, Gendaszek AE (2002). Illicit use of psychostimulants among college students: a preliminary study. Psychology, Health & Medicine.

[B24] Diefenbach GJ, Diefenbach D, Baumeister A, West M (1999). Portrayal of lobotomy in the popular press: 1935–1960. Journal of the History of the Neurosciences.

[B25] Racine E, Waldman S, Palmour N, Risse D, Illes J (2007). "Currents of hope": neurostimulation techniques in U.S. and U.K. print media. Cambridge Quarterly of Healthcare Ethics.

[B26] Decety J, Jackson PL, Sommerville JA, Chaminade T, Meltzoff AN (2004). The neural bases of cooperation and competition: an fMRI investigation. NeuroImage.

[B27] Rose SPR (2003). How to (or not to) communicate science. Biochemical Society Transactions.

[B28] Racine E, Bar-Ilan O, Illes J (2006). Brain Imaging: a decade of coverage in the print media. Science Communication.

[B29] Babcock Q, Byrne T (2000). Student perceptions of methylphenidate abuse at a public liberal arts college. Journal of American College Health.

[B30] Racine E, Bar-Ilan O, Illes J (2005). fMRI in the public eye. Nature Reviews Neuroscience.

[B31] Racine E, Gareau I, Doucet H, Laudy D, Jobin G, Schraedley-Desmond P (2006). Hyped biomedical science or uncritical reporting? Press coverage of genomics (1992–2001) in Québec. Social Science & Medicine.

[B32] Neuendorf KA (2002). The Content Analysis Guidebook.

[B33] Adams L (2005). How Ritalin became poor man's cocaine; warnings over increasing adult abuse of children's drug.

[B34] Diaz J (2001). Ritalin grows as 'cramming drug' at U.S. colleges; medicine can have serious side effects.

[B35] Garreau J (2006). 'Smart pills' are on the rise. But is taking them wise?.

[B36] Lite J (2006). Who needs a dealer...when you have a doctor? New Yorkers are just saying yes to prescription abuse.

[B37] Phillips S (2006). An espresso in the morning is just so last year.

[B38] Reinink A (2001). Colleges eye ways to curb Ritalin abuse.

[B39] Zernike K (2005). The difference between steroids and Ritalin is....

[B40] Hall W (2004). Feeling 'better than well'. EMBO Reports.

[B41] Teter CJ, McCabe SE, LaGrange K, Cranford JA, Boyd CJ (2006). Illicit use of specific prescription stimulants among college students: prevalence, motives, and routes of administration. Pharmacotherapy.

[B42] Novartis (2007). Ritalin, Product information.

[B43] Novartis (2008). Ritalin LA, Product information.

[B44] Novartis (2007). Ritalin, Product information.

[B45] Novartis (2007). Ritalin, Product information.

[B46] Zeilbauer P (2000). New Campus High: Illicit Prescription Drugs.

[B47] Khan M (2003). Study drugs draw concern.

[B48] Mehlman MJ (2004). Cognition-enhancing drugs.

[B49] Barrett SP, Darredeau C, Bordy LE, Pihl RO (2005). Characteristics of methylphenidate misuse in a university student sample. Can J Psychiatry.

[B50] Hupp S (2006). Students abusing ADHD drugs: prescription pills easy to get, youths, health workers say.

[B51] Teter CJ, McCabe SE, Cranford JA, Boyd CJ, Guthrie SK (2005). Prevalence and motives for illicit use of prescription stimulants in an undergraduate student sample. Journal of American College Health.

[B52] White BP, Becker-Blease KA, Grace-Bishop K (2006). Stimulant medication use, misuse, and abuse in an undergraduate and graduate student sample. Journal of American College Health.

[B53] Whitehouse PJ, Juengst E, Mehlman M, Murray TH (1997). Enhancing cognition in the intellectually intact. Hastings Centre Report.

[B54] Anonymous (2004). A new form of drug abuse.

[B55] Rajczi A (2008). One danger of biomedical enhancements. Bioethics.

[B56] Lanni C, Lenzken SC, Pascale A, Del Vecchio I, Racchi M, Pistoia F, Govoni S (2008). Cognition enhancers between treating and doping the mind. Pharmacological Research.

[B57] Elliott R, Sahakian BJ, Matthews K, Bannerjea A, Rimmer J, Robbins TW (1997). Effects of methylphenidate on spatial working memory and planning in healthy young adults. Psychopharmacology.

[B58] Mehta MA, Owen AM, Sahakian BJ, Mavaddat N, Pickard JD, Robbins TW (2000). Methylphenidate enhances working memory by modulating discrete frontal and parietal lobe regions in the human brain. Journal of Neuroscience.

[B59] Barch DM, Carter CS (2005). Amphetamine improves cognitive function in medicated individuals with schizophrenia and in healthy volunteers. Schizophrenia Research.

[B60] Bray CL, Cahill KS, Oshier JT, Peden CS, Theriaque DW, Flotte TR, Stacpoole PW (2004). Methylphenidate does not improve cognitive function in healthy sleep-deprived young adults. Journal of Investigational Medicine.

[B61] Farah MJ, Haimm C, Sankoorikal G, Chatterjee A (2008). When we enhance cognition with Adderall, do we sacrifice creativity? A preliminary study. Psychopharmacology.

[B62] Canadian Pharmacists Association (2008). Ritalin. e-Compendium of Pharmaceuticals and Specialties Toronto.

[B63] Maher B (2008). Poll results: look who's doping. Nature.

[B64] Coveney CM, Nerlich B, Martin P (2008). Modafinil in the media: metaphors, medicalisation and the body. Social Science & Medicine.

[B65] Kramer P (1997). Listening to Prozac.

[B66] Greely H, Sahakian B, Harris J, Kessler RC, Gazzaniga M, Campbell P, Farah MJ (2008). Towards responsible use of cognitive-enhancing drugs by the healthy. Nature.

[B67] Australian Press Council (2001). Reporting guidelines: drugs and drug addiction.

[B68] Olshansky SJ, Perls TT (2008). New developments in the illegal provision of growth hormone for "anti-aging" and bodybuilding. Journal of the American Medical Association.

[B69] Chatterjee A (2004). Cosmetic neurology: the controversy over enhancing movement, mentation, and mood. Neurology.

[B70] National Institute on Drug Abuse (2005). Prescription drugs: abuse and addiction.

[B71] Bergstrom LS, Lynoe N (2008). Enhancing concentration, mood and memory in healthy individuals: an empirical study of attitudes among general practitioners and the general population. Scandinavian Journal of Public Health.

