# Supplementary material for: Disagreements with implications: diverging discourses on the ethics of non-medical use of methylphenidate for performance enhancement
Source: BMC Med Ethics. 2009 Jul 6;10:9. doi: 10.1186/1472-6939-10-9 (PMC2719652; doi:10.1186/1472-6939-10-9)
Supplement: Additional file 1 — Coding structure used to analyze media, bioethics, and public health discourses on the non-medical use of methylphenidate (MPH). [file 1472-6939-10-9-S1.doc]

Additional File 1

Coding structure used to analyze media, bioethics, and public health discourses on the non-medical use of methylphenidate (MPH)

| *Section 1: Description of non-medical use of MPH* | | |
| --- | --- | --- |
| 1.1. Definitions and synonyms for non-medical use of MPH | | |
|  | Formal definition of non-medical use of MPH | |
|  | Lay descriptions of non-medical use of MPH | |
|  | Distinction between treatment and enhancement | |
| 1.2. Uses of MPH | | |
|  | Medical use | |
|  | Cognitive and academic performance enhancement use | |
|  | Recreational use | |
| 1.3. Extent of non-medical use of MPH | | |
|  | Accepted | |
|  | Frequent | |
|  | Neutral | |
|  | Questionable | |
|  | Rare | |
| 1.4. Description of practices of non-medical use of MPH | | |
|  | When is MPH used non-medically | |
|  | Who is using MPH non-medically | |
|  | Where is MPH used non-medically | |
|  | How MPH is procured for non-medical use | |
|  | | Black market |
|  | | Buying pills from other students |
|  | | Feigning symptoms of ADHD |
|  | | Online pharmacies |
|  | | Other |
| *Section 2: Workings and effects of MPH* | | |
| 2.1. Physiological effects of non-medical use of MPH | | |
|  | Physiological negative effects of non-medical use of MPH | |
|  | Physiological positive effects of non-medical use of MPH | |
| 2.2. Psychological effects of non-medical use of MPH | | |
|  | Psychological negative effects of non-medical use of MPH | |
|  | Psychological positive effects of non-medical use of MPH | |
| 2.3. Unknown effects of non-medical use of MPH | | |
| *Section 3: Ethical, social and legal issues associated with non-medical use of MPH ** | | |
| 3.1. Social integration and acceptability | | |
| 3.2. Social meaning | | |
| 3.3. Unsafe | | |
| 3.4. Abuse | | |
| 3.5. Cheating | | |
| 3.6. Inauthenticity, identity, and personhood | | |
| 3.7. Injustice, access, and equality | | |
| 3.8. Overprescription | | |
| 3.9. Lack of autonomy, individual rights, and informed consent | | |
| 3.10. Illegality | | |
| 3.11. Commercialization | | |
| 3.12. Inefficacy | | |
| *Section 4: Prevention of non-medical use of MPH and related challenges* | | |
| 4.1 Solutions | | |
| 4.2 Challenges | | |
| *Content under these codes was further analyzed into 3 coding options: (1) affirmation of the issue; (2) negation of the issue; (3) neutral or ambivalent stance regarding the issue | | |
